# Prenatal Diagnosis of Renal Anomalies Associated With a Novel Causative Variant in RAP1B Gene

**DOI:** 10.1002/ccr3.72277

**Published:** 2026-03-13

**Authors:** Adalgisa Cordisco, Stefania Magliulo, Chiara Di Marco, Elisa Fortuna, Alessandra Terracciano, Claudio Meloni

**Affiliations:** ^1^ Division of Prenatal Diagnosis P. Palagi and San Giovanni di Dio Hospital Florence Italy; ^2^ Division of Prenatal Diagnosis Azienda Ospedaliero Universitaria Meyer Florence Italy; ^3^ Medical Genetics Laboratory, Translational Cytogenomics Research Unit Bambino Gesù Children's Hospital, IRCCS Rome Italy; ^4^ Department of Biomedicine and Prevention Tor Vergata University of Rome Rome Italy; ^5^ Division of Medical Genetics, Azienda USL Toscana Centro Santa Maria Nuova Hospital Firenze Italy; ^6^ Obstetric and Prenatal Medicine Unit IRCCS Azienda Ospedaliero‐Universitaria Sant'orsola‐Malpighi Bologna Italy; ^7^ Division of Obstetrics and Gynecology San Giovanni di Dio Hospital Florence Italy

**Keywords:** prenatal diagnosis, RAP1B gene, renal agenesis, renal cystic dysplasia

## Abstract

A detailed description of prenatal ultrasound signs of congenital renal cystic dysplasia (CRCD) is reported. Molecular investigations identified the c.179G>T, p.(Gly60Val) “de novo” variant in a heterozygous state in the RAP1B gene. This is a missense variant not described in the literature. Predictive tools suggest a pathogenic role for this mutation and a likely association with the clinical phenotype.

## Graphical abstract

A case of prenatal renal cystic disease and unilateral renal agenesis is reported. Molecular investigations including TRIO next‐generation sequencing analysis using the Clinical exome sequencing protocol identified the c.179G>T, p.(Gly60Val) “de novo” variant in a heterozygous state in the RAP1B gene. The mutation in the RAP1B gene suggests a likely association with the clinical phenotype.

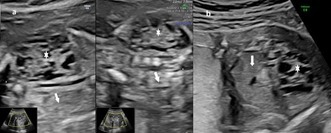

## 1 Introduction

Unilateral renal agenesis (URA) is defined as the complete absence of one kidney and ipsilateral ureter due to either the failure of the development of the ureteric bud in forming the ureter, renal pelvis, and renal interstitium, or a defect in its interaction with the metanephric blastema, involving just one side of the urinary tract [1, 2]. Renal cystic disease encompasses a complex group of pathologic and clinical entities, with varied but distinctive sonographic features. There are many varieties of CRCD, and ultrasound enables a specific diagnosis in only a few cases. The two key features are: non‐confluent cystic formations variably distributed within the parenchyma, or marked echogenicity, typically reflecting the presence of diffuse microcysts [3]. As a frequent form of congenital abnormality, URA and renal cystic disease can be diagnosed during pregnancy. After fetal instrumental diagnosis, the couple is usually offered targeted molecular investigations for detection of possible genetic causes involved and subsequent counseling. The following case describes a novel causative variant in the RAP1B gene classified as “pathogenic” associated with complex fetal urinary abnormalities.

## 2 Case History

**FIGURE 1:**
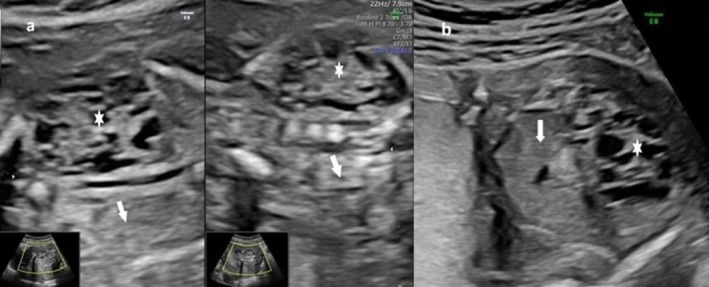
Coronal (a) and transverse (b) ultrasound view of a fetus at 20 weeks of gestational age showing the absence of the right kidney in its *loggia* (arrows) and the concomitant presence of cystic dysplasia in the left kidney (asterisk symbol).

A twenty‐three‐year‐old *primigravida* was referred to our prenatal diagnosis center after the detection of anhydramnios at a peripheral hospital during the eighteenth week of gestation. She reported a normal first‐trimester ultrasound. Her personal and family medical history was unremarkable. Ultrasound examination revealed reduced fetal movements and anhydramnios, secondary to left CRCD and concomitant right renal agenesis (no renal tissue or renal artery were visualized in the right renal *loggia*) (shown in Figure 1). Normal kidney tissue (parenchyma) was not detectable by ultrasound because it was replaced by nonfunctioning fibrous tissue organized into cysts of various sizes, with a maximum diameter of less than 1 cm and negative to color Doppler, which were not connected to each other or to the urinary collection system (renal pelvis and ureter) (Figure 1). The size of the dysplastic kidney was slightly increased for gestational age (longitudinal × transverse × anteroposterior diameter, 24 × 15 × 18 mm). The bladder walls were persistently collapsed due to the lack of amniotic fluid production. The remaining anatomy appeared normal, with biometry at the lower limits for gestational age, consistent with 17 weeks.

## 3 Differential Diagnosis

The main differential diagnosis of URA is renal ectopia, that is, the presence of renal parenchyma in an atypical location, usually pelvic. In early gestation, the kidney has a very similar echogenicity to that of the bowel, making it difficult to distinguish between them clearly.

The main differential diagnoses of CRCD include other conditions that cause cysts in the kidneys or that may appear similar on diagnostic imaging (ultrasound). The most important ones are described below.

Multicystic dysplastic kidney (MCDK) is characterized by abnormal and disorganized development of renal tissue (parenchyma). The kidney is replaced by multiple, noncommunicating cysts of varying sizes arranged in a disorganized manner, which obliterate the normal renal collecting system (renal calyces and pelvis) [4]. The kidney is nonfunctional or severely hypofunctional. The main cause is believed to be complete or near‐complete obstruction of the mesonephric duct (primitive ureter) during fetal renal organogenesis, which prevents proper interaction between the metanephric blastema and the ureteric bud. In most cases, the condition is unilateral.

Autosomal recessive polycystic kidney disease (ARPKD) is characterized by bilaterally enlarged, echogenic kidneys containing diffuse microcysts, often leading to early renal failure and hepatic fibrosis [5]. It differs from CRCD (usually unilateral, with disorganized macrocysts and absence of functioning parenchyma) in its bilateral nature, the size of the cysts, and its association with liver disease.

Autosomal dominant polycystic kidney disease (ADPKD): later onset, characterized by multiple cysts that develop progressively in both kidneys, with progressive deterioration of kidney function [5]. It rarely occurs in the neonatal period in such a severe form that it can be confused with ‐CRCD. Family history is a key diagnostic factor.

Severe obstructive hydronephrosis: severe dilation of the renal collecting system due to mechanical obstruction (e.g., stenosis of the ureteropelvic junction). On ultrasound, dilated calyces may mimic cysts. The crucial distinction is the demonstration of communication between the dilated cavities and the renal pelvis, which does not occur in CRCD. The use of Doppler can help identify residual parenchyma and preserved vascular flow, unlike in CRCD where the renal tissue is disorganized and hypovascularized.

Cystic renal tumors (e.g., cystic nephroblastoma, multicystic renal carcinoma): although rare in children, some neoplasms may present with a predominant cystic component. CRCD is usually characterized by an absence of solid components within the cysts or tumor vascular architecture. Advanced imaging (MRI) may be necessary to rule out malignancy.

Acquired simple or multilocular renal cysts: generally isolated, benign, and not associated with dysgenesis of the surrounding renal tissue.

## 4 Conclusion and Results

**FIGURE 2:**
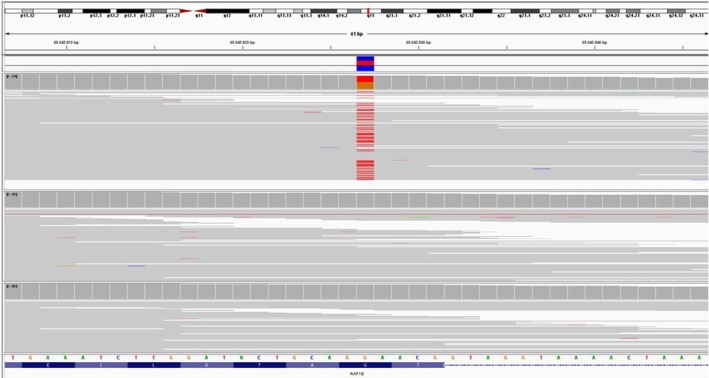
NGS analysis unveiling the NM_001010942.3 (RAP1B): C.179G>T, p.(Gly60Val) variant of de novo origin in the fetus.

After obtaining written informed consent for molecular analysis, the patient underwent an invasive chorionic villus sampling. The karyotype was normal (46,XX). Array‐CGH analysis did not reveal any microdeletions or microduplications in the female genomic profile. The couple subsequently opted to pursue further diagnostic investigations through a clinical exome sequencing (CES) approach. This analysis was conducted on DNA extracted from chorionic villi and peripheral blood DNA from both partners. Variants identified by sequencing analysis were prioritized based on clinical signs revealed by ultrasound. The investigation identified the c.179G>T, p.(Gly60Val) variant in a heterozygous state in the RAP1B gene, which was determined to be “de novo” in origin, as it was not present in the DNA of either parent (shown in Figure 2). This is a missense variant not reported in the general population database gnomAD, not annotated in the ClinVar database, and not described in the literature. The couple decided to terminate the pregnancy at 20 weeks. Macroscopic evaluation during autopsy revealed: agenesis of the right kidney with the presence of the adrenal gland in situ, absence of the right ureter; enlarged left kidney with an altered profile due to the presence of numerous cysts of various sizes and a “spongy” appearance. Microscopic evaluation revealed a left renal parenchyma edematous and disorganized, with cystic and dilated tubules of varying sizes, some surrounded by immature mesenchyme, immature tubules, and a few abortive glomeruli.

## 5 Discussion

URA can be diagnosed during pregnancy. X‐linked, autosomal dominant, and recessive inheritance have been reported for renal agenesis [6]. At present, the suspected pathogenic genes reported include WT1, EYA1, SIX1, and LIM1, with most playing an important role in the signal transduction of kidney development [7]. In Yang H. study [8], trio‐WES and CNV‐seq were used to examine genetic pathogenic factors of URA in multiethnic children. A total of three variants were identified in three of the ten patients in relation to three genes: CHD7, PROKR2, and NRIP1. All variants were missense and in the heterozygous state. Renal cystic disease encompasses a complex group of pathological and clinical entities characterized by the presence of non‐confluent cystic formations within the parenchyma and marked renal echogenicity [3]. The etiology of isolated renal dysplasia is multifactorial in the majority of cases. It may occur as an isolated condition or as a component of numerous rare syndromes, such as Meckel syndrome, Joubert syndrome, Brachio‐otorenal syndrome, Bardet‐Biedl syndrome, and 17q12 deletion syndrome, etc. Chromosomal abnormalities and syndromes are present in approximately 7%–14% of pregnancies with suspected multicystic dysplastic kidney.

The c.179G>T, p.(Gly60Val) variant in the heterozygous state in the RAP1B gene is a missense variant not reported in the general population database gnomAD, not annotated in the ClinVar database, and not described in the literature. Predictive tools suggest a pathogenic role for this mutation. A different amino acid change at the same position is a known pathogenic variant. RAP1B is a member of the RAS superfamily of small GTPases, which are involved in many cellular processes [9]. Pathogenic variants in the RAP1B gene are associated with thrombocytopenia 11 with multiple congenital anomalies and dysmorphic facies (OMIM #620654). This condition follows an autosomal dominant inheritance pattern and is clinically characterized by a variable combination of distinctive facial features, chronic and persistent thrombocytopenia, postnatal microcephaly, hypotonia, intellectual disability with learning difficulties, and multiple congenital anomalies. These anomalies may involve cardiac, cerebral, skeletal, and genitourinary systems, including renal agenesis and cystic renal dysplasia. The cases described in literature are very limited, and even less is known about potential prenatal manifestations of this condition. Niemann et al. [10] identified de novo variants in the RAP1B gene (c.35G>T p.(Gly12Val) and c.178G>C p.(Gly60Arg)) in two unrelated patients with thrombocytopenia, microcephaly, learning difficulties, renal malformations, structural anomalies of the brain and other features. Pardo et al. [9] identified heterozygous variants in RAP1B: c.35G>A, p.(Gly12Glu) and c.178G>A, p.(Gly60Arg) in two unrelated individuals with thrombocytopenia, congenital malformations, and neurological, behavioral, and dysmorphic features.

The substitution is predicted to be deleterious by CADD (Phred 31.00), REVEL (0.96), PolyPhen‐2 (0.993), and AlphaMissense (0.9989). Based on data available in the literature and ACMG guidelines, the identified variant can be classified as “pathogenic” by the application of the following criteria: PM1_Moderate, PM2_Supporting, PM5_Moderate, PP2_Supporting, PP3_Supporting, and PS2_Strong. It can be hypothesized that this variant may represent the cause of the renal abnormalities observed in the fetus and that further clinical manifestations might have become evident after birth. Further functional studies and identification of additional affected patients may help clarify the clinical variability of syndromes associated with RAP1B. In conclusion, the role of the missense variant c.179G>T, p.(Gly60Val) in heterozygosity in the RAP1B gene, not reported in scientific literature, suggests a likely association with the clinical phenotype.

## Author Contributions

**Adalgisa Cordisco:** conceptualization, data curation, writing – original draft. **Stefania Magliulo:** investigation, methodology. **Chiara Di Marco:** investigation, methodology. **Elisa Fortuna:** writing – original draft. **Alessandra Terracciano:** investigation, methodology. **Claudio Meloni:** writing – review and editing.

## Funding

The authors have nothing to report.

## Ethics Statement

The research was conducted ethically in accordance with the World Medical Association Declaration of Helsinki. The patient has given her written informed consent to publish her case (including publication of images). Ethical approval was not required for this study in accordance with local guidelines.

## Conflicts of Interest

The authors declare no conflicts of interest.

## Data Availability

Data sharing not applicable to this article as no datasets were generated or analyzed during the current study.
